# Chlorine Inactivation of *Elizabethkingia* spp. in Water

**DOI:** 10.3201/eid3010.240917

**Published:** 2024-10

**Authors:** David A. Holcomb, Diana Riner, Benjamin Cowan, Zainab Salah, Wiley C. Jennings, Mia C. Mattioli, Jennifer L. Murphy

**Affiliations:** Centers for Disease Control and Prevention, Atlanta, Georgia, USA

**Keywords:** Disinfection, halogenation, biofilms, Flavobacteriaceae, opportunistic infections, disease outbreaks, Drug Resistance, bacteria, water, water supply, sanitation, environmental microbiology, chlorine, *Elizabethkingia*, antimicrobial resistance

## Abstract

We performed chlorine inactivation experiments for *Elizabethkingia anophelis* and *E. meningoseptica* bacterial strains from clinical and environmental sources. Free chlorine concentration × contact time values <0.04 mg·min/L achieved 99.9% inactivation of *Elizabethkingia* species, indicating chlorine susceptibility. Measures to control biofilm producing pathogens in plumbing are needed to prevent *Elizabethkingia* bacterial infections.

*Elizabethkingia* spp. are widely distributed environmental bacteria and opportunistic pathogens that can cause sepsis and meningitis, particularly in neonates (*1*,*2*). At least 3 phenotypically similar species (*E. anophelis*, *E. meningoseptica*, and *E. miricola*) are intrinsically resistant to multiple antimicrobial classes and have been implicated in fatal healthcare-associated outbreaks (*3*). The largest reported United States outbreak was an *E. anophelis* strain that caused 66 laboratory-confirmed infections in Wisconsin and neighboring states in 2015–2016, for which the infection source was never identified. That outbreak was unusual for primarily consisting of community-acquired infections (*4*). Plumbing fixtures such as taps and sink drains, which *Elizabethkingia* bacteria readily colonize in biofilms, are common exposure vehicles in healthcare settings (*2*,*5*,*6*). Transmission from handwashing in contaminated sinks is of particular concern and has been shown to contaminate health worker hands with *E. anophelis*, even with the use of chlorhexidine soap (*5*).

Chlorination is the most common disinfection method for public water supplies. When used at the end of the treatment chain, chlorination provides residual disinfection during distribution and storage. Reports of *Elizabethkingia* bacterial persistence in chlorinated water supplies and plumbing fixtures cleaned with sodium hypochlorite have raised concerns of chlorine tolerance (*2*,*6*,*7*), but no data have been published. We conducted disinfection experiments with a free chlorine residual (FCR) dose of 0.2 mg/L, the minimum disinfectant residual for treated surface water entering distribution systems in the United States, to assess inactivation of 2 *Elizabethkingia* spp. isolated from clinical and environmental samples (*8*). We fit inactivation kinetics models to estimate the product of FCR dose (C; mg/L) and contact time (T; minutes) required to reduce *Elizabethkingia* bacterial concentrations by 99.9% (CT_99.9%_).

## The Study

We performed disinfection experiments in triplicate for 6 *E. anophelis* and 5 *E. meningoseptica* strains from clinical and environmental sources (Table 1). We prepared bacterial stocks by incubating cultures in tryptic soy broth overnight at 37°C, followed by subculture into tryptic soy broth at 37°C for ≈5 hours (*9*). We pelleted the log phase cultures, washed with sterile phosphate-buffered saline (PBS), repelleted, and resuspended in 5 ml PBS. We prepared 50-mL glass flasks with 25 mL of sterile oxidant demand–free water buffered at pH 7.5, dosed to 0.2 mg/L FCR with 5.25% sodium hypochlorite, and maintained in a water bath at 25°C. We seeded flasks with 0.1 mL bacterial stock and extracted 10 mL aliquots from 3 flasks after 15, 30, and 60 seconds, immediately quenching the aliquots with 100 µL of 10% sodium thiosulfate (Fisher Scientific, https://www.fishersci.com). We extracted triplicate aliquots from 3 chlorine-free flasks at 60 seconds to examine dieoff with no disinfectant exposure. We also measured FCR in aliquots removed at 30 and 60 seconds from a final seeded flask to assess disinfectant decay. We serially diluted the initial bacterial stocks and experimental aliquots with sterile PBS and enumerated by using membrane filtration plated onto tryptic soy agar containing 5% rabbit blood, incubated at 37°C, and counted after 24–36 hours.

**Table 1:** *Elizabethkingia* strains from environmental and clinical sources provided by the Centers for Disease Control and Prevention used for study of chlorine inactivation of *Elizabethkingia* spp. in water

Species	Strain	Accession no.	Year	Location	Source	Origin
*E. anophelis*	DSM 23781	FLST00000000	2009	The Gambia	Insect*	Midgut
	CSID_3015183678	MAFY00000000	2016	Wisconsin, USA	Clinical	Blood
	CSID_3015183679	MAHO00000000	2016	Wisconsin, USA	Clinical	Blood
	CSID_3015183681	GCA_001618545.2	2016	Wisconsin, USA	Clinical	Blood
	17-336	NA	2017	Oklahoma, USA	Environmental	Sink drain swab
	17-337	NA	2017	Oklahoma, USA	Environmental	Sink drain swab
*E. meningoseptica*	KC1913 (ATCC 13253)	LNOH00000000	1959	Massachusetts, USA	Clinical	Spinal fluid
	16-062	NA	2016	Minnesota, USA	Clinical	Bronchial wash
	16-148	NA	2016	Florida, USA	Clinical	Blood
	17-276	NA	2016	Michigan, USA	Clinical	Sputum
	2016-08-103-03	NA	2016	NA	Environmental	NA

*DSM 23781 was isolated from the *Anopheles gambiae* mosquito midgut and was excluded from comparisons between clinical and environmental sources. NA, not available.

We analyzed inactivation kinetics by modeling the natural log-survival, the ratio of the bacterial concentration at contact time T to the initial concentration, as a function of time and FCR dose governed by an inactivation rate constant *k*. We estimated *k* by using the pseudo-first order Chick-Watson model and the nonlinear generalization, the Hom model, assuming first-order disinfectant decay and excluding samples for which the concentration was too low to detect (*10*). We used R version 4.3.2 (The R Project for Statistical Computing, https://www.R-project.org) to fit inactivation models by nonlinear least squares; estimate disinfectant decay rates, *k’*, with linear regression; calculate CT values from the fitted kinetic models; and perform z-tests (5% significance level) to compare rate constant estimates between species and between strain sources (*11*,*12*). Both inactivation kinetic models provided comparable fits to the experimental data that were indicated by similar values of the Akaike information criterion and root mean square error (Table 2). However, the Hom model was computationally unstable and produced rate constant estimates with larger SEs. Because of the larger SEs, we used only the Chick-Watson model to compare rate constants of different species and between clinical and environmental strains. Sensitivity analyses that accounted for potential correlation between replicates by using the geometric mean concentration to calculate strain-specific log-survival at each time point produced comparable rate constant estimates and CT values.

**Table 2:** Inactivation kinetic model performance metrics, rate constant estimates, and disinfectant CT values needed to achieve a 99%–99.99% reduction in *Elizabethkingia* detection in study of chlorine inactivation of *Elizabethkingia* spp. in water*

Comparison	N	No. (%) ND	AIC	RMSE	Rate constant (SE),† min^–1^		Pooled SE‡	z-score (p value)	CT value, mg·min/L		
*k’*	ln(*k*)	99%	99.9%	99.99%
Model, all data											
Chick-Watson	83	21 (20)	430	3.1	0.89 (0.08)	10.4 (1.3)	NA	NA	0.021	0.031	0.042
Hom			431	3.0		22.1 (18.4)			0.021	0.026	0.029
Species, Chick-Watson model											
* E. anophelis*	35	15 (30)	183	3.0	0.90 (0.13)	10.3 (1.8)	2.5	0.10 (0.92)	0.019	0.029	0.039
* E. meningoseptica*	48	6 (11)	248	3.0	0.88 (0.11)	10.0 (1.8)			0.023	0.035	0.047
Source,§ Chick-Watson model											
Clinical	51	18 (26)	280	3.5	0.90 (0.09)	10.0 (1.9)	2.2	0.90 (0.37)	0.021	0.032	0.043
Environmental	27	0 (0)	120	2.0	1.09 (0.13)	8.1 (1.1)			0.025	0.037	0.050

*AIC, Akaike information criterion; CT value, product of concentration and contact time required to achieve specified reduction; N, number of observations (out of 104 total) with detectable *Elizabethkingia* used to fit model; ND, not detected; RMSE, root mean square error. †Point estimates and SEs of the disinfectant decay rate constant *k’* and natural logarithm of the inactivation rate constant *k*. ‡Pooled SE for z-test of the equality of rate constants for each subset, obtained as the square root of the sum of the squared standard errors of both rate constant estimates. §The *E. anophelis* type strain DSM 23781 was isolated from an insect host and thus excluded from the comparison of clinical and environmental sources.

Both models indicated a free chlorine CT of ≈0.03 milligram-minutes per liter (mg·min/L) inactivated 99.9% of *Elizabethkingia* bacteria (Table 2). In stratified Chick-Watson analyses, both species demonstrated CT_99.9%_ <0.04 mg·min/L and rate constant estimates that were not statistically different (p = 0.92). However, *E. anophelis* was reduced to undetectable concentrations at 1 minute of exposure in 30% (n = 15) of samples, whereas *E. meningoseptica* was not detected at 1 minute of exposure in only 11% (n = 6) of samples. Environmental strains displayed less variable log-survival than clinical strains (Figure), but the rate constant estimates were not statistically different (p = 0.37). Both strains produced similar CT_99.9%_ values of 0.04 mg·min/L (environmental) and 0.03 mg·min/L (clinical). All environmental strains were still detectable after 1 minute of exposure, whereas clinical strains were not detected in 18 samples (26%).

**Figure:**
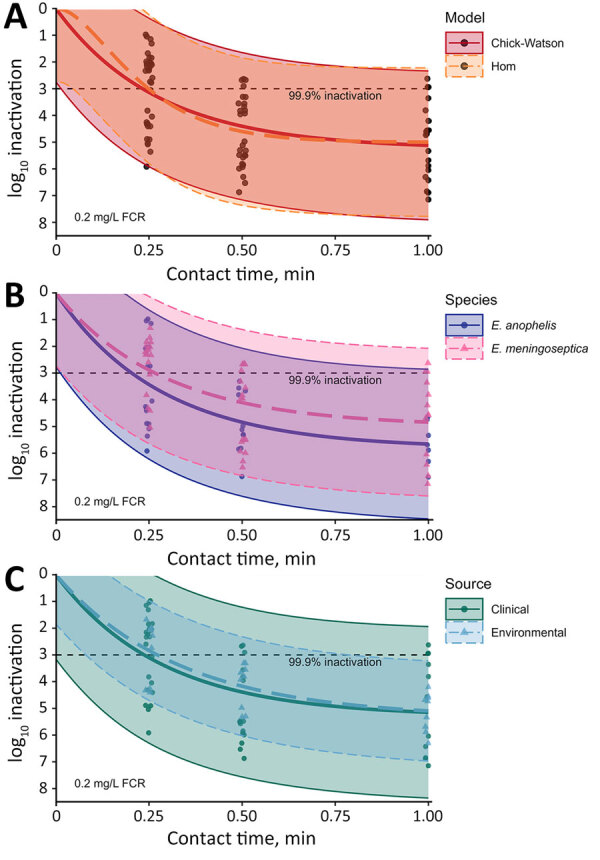
Observed and model-predicted log_10_ inactivation in study of chlorine inactivation of *Elizabethkingia* spp. in water. Samples from environmental and clinical sources were exposed to chlorine in water with increasing contact time at an initial dose of 0.2 mg/L FCR. Panels show comparisons in modeled inactivation by model specification. A) All data used for both models. B) Stratified by species, Chick-Watson model. C) Strain source, Chick-Watson model. Curved lines indicate model-predicted inactivation; dots, observed inactivation; and shaded regions, model 95% prediction intervals. FCR, free chlorine residual.

The initial FCR dose was reduced by approximately two thirds after 1 minute. The median reduction in *Elizabethkingia* after 1 minute with no chlorine exposure was 0.13 log_10_ (26%). In contrast, the Chick-Watson model predicted ≈5 log_10_ (99.999%) inactivation in 1 minute for a 0.2 mg/L FCR dose (Figure). Model 95% prediction intervals indicated a minimum expected inactivation of ≈2 log_10_ after 1 minute at the experimental conditions (Figure). Of the 1-minute samples, >3 log_10_ inactivation was observed for 32 of the 35. The 3 samples below the 3 log_10_ inactivation threshold were replicates of the same clinical strain and experienced 2.6–2.9 log_10_ inactivation.

## Conclusions

Contrary to the chlorine tolerance hypothesized in the literature, we observed rapid inactivation of *Elizabethkingia* at typical point-of-use free-chlorine concentrations (≈0.2 mg/L). Across species and sources, *Elizabethkingia* strains demonstrated greater chlorine susceptibility (CT_99.9%_; <0.04 mg·min/L) than a reported *Escherichia coli* reference strain (CT_99.9%_; 0.09 mg·min/L) that was used to benchmark disinfectant susceptibility of waterborne pathogens (*13*). However, we also observed cells persisting at detectable concentrations after 1 minute of contact time, particularly among environmental strains. The more persistent subpopulations could seed biofilms, which *Elizabethkingia* bacteria readily form in plumbing fixtures, and have been shown to rapidly recolonize sink drains within days of seemingly effective disinfection (*2*,*14*), possibly accounting for the reported survival of *Elizabethkingia* spp. after chlorination in healthcare settings. Biofilms can protect embedded organisms from disinfection through multiple mechanisms, including oxidant demand exerted by the extracellular matrix, limited diffusion of the disinfectant to inner layers, and phenotypic adaptations in response to sublethal disinfectant doses and the biofilm environment itself (*15*). A review of 6 bacteria species reported biofilm-embedded cells required 2–600 times the chlorine dose or contact time for inactivation than their planktonic (free-swimming) counterparts (*15*). Prevention of *Elizabethkingia* infections, as with other opportunistic biofilm pathogens, may be most readily accomplished by limiting the environments in which biofilms can form and reducing exposure to potentially contaminated sources (*5*–*7*). Building managers should adopt water management programs to limit the growth and transmission of opportunistic pathogens of plumbing.
